# DELUSION OF PREGNANCY IN PATIENT WITH MAJOR NEUROCOGNITIVE DISORDER: A CASE REPORT

**DOI:** 10.1192/j.eurpsy.2023.1990

**Published:** 2023-07-19

**Authors:** M. O. Pires, S. Mouta, I. Fonseca Vaz, B. Jesus, J. Nunes, A. Pissarra da Costa

**Affiliations:** 1 Departamento de Psiquiatria e Saúde Mental, ULS Guarda, Guarda, Portugal

## Abstract

**Introduction:**

Delusion of pregnancy (DP) is a heterogeneous symptom that can emerge from different neuropsychiatric syndromes, including schizophrenia, bipolar disorders, but also major neurocognitive disorder (MND). According to the Diagnostic and Statistical Manuel of Mental Disorders-5 (DSM-5), DP is an unspecified type of delusional disorder present in the spectrum of schizophrenia and other psychotic disorders This type of delusion, which can affect both sexes, may have numerous determinants to its genesis and may last decades to resolve.

**Objectives:**

We aim to present a case and review of DP and its association with dementia/MND, hyperprolactinemia and galactorrhea.

**Methods:**

Non-systematic literature review and case report, based on the search for titles and/or abstracts of articles that address both DP and dementia, and DP and hyperprolactinemia/galactorrhea, including articles published between 2010 and 2022 in English.

**Results:**

A 71-year-old female patient was admitted to the Psychiatric unit due to a change in usual behavior in the past 6 months: insomnia, anterograde amnesia, delusions of ruin and persecutory and, for the past month, the belief of being pregnant with twins, supported by the galactorrhea she presented after starting Risperidone prescribed by her Family Doctor weeks prior. Shortly after admission, the patient also revealed hearing her fetuses’ voices. DP vanished briefly after admission due to the combination between the change of Risperidone to Aripiprazole (a prolactin-sparing antipsychotic) and psychotherapy to help deconstruct the patient’s cognitive misinterpretations. She was furthermore diagnosed with Alzheimer’s disease and Memantine was started.

**Conclusions:**

This patient, according to Bera et al. (Bera *et.al.* Indian J Psychol Med 2015;37:131-7) is part of the 28.6% of patients more than 50 years of age who present DP, 6.0% that report having twins and 8.3% that report hearing voices of their fetuses. No data was found correlating DP and MND directly. Hyperprolactinemia and its consequent galactorrhea represent one of the many explanations behind DP, especially in suggestible demented patients that easily misinterpret somatic sensations, in which delusional thoughts are frequent and contribute to the morbidity.

**Disclosure of Interest:**

None Declared

